# Survival Outcomes of Patients with Pathologically Proven Positive Lymph Nodes at Time of Radical Cystectomy with or without Neoadjuvant Chemotherapy

**DOI:** 10.3390/jcm9061962

**Published:** 2020-06-23

**Authors:** Guillaume Ploussard, Benjamin Pradere, Jean-Baptiste Beauval, Christine Chevreau, Christophe Almeras, Etienne Suc, Jean-Romain Gautier, Anne-Pascale Laurenty, Mathieu Roumiguié, Guillaume Loison, Christophe Tollon, Loïc Mourey, Ambroise Salin, Evanguelos Xylinas, Damien Pouessel

**Affiliations:** 1 Department of Urology, La Croix du Sud Hospital, 31130 Quint Fonsegrives, France; jbbeauval@gmail.com (J.-B.B.); c.almeras@gmail.com (C.A.); gautierjr@hotmail.fr (J.-R.G.); guillaumeloison@gmail.com (G.L.); tol@club-internet.fr (C.T.); ambroise.salin@gmail.com (A.S.); 2 Department of Urology, Bretonneau Hospital, 37000 Tours, France; benjaminpradere@gmail.com; 3 Department of Urology, Comprehensive Cancer Center, Medical University of Vienna, 1090 Vienna, Austria; 4 Department of Oncology, IUCT-O, 31000 Toulouse, France; chevreau.christine@iuct-oncopole.fr (C.C.); mourey.loic@iuct-oncopole.fr (L.M.); pouessel.damien@iuct-oncopole.fr (D.P.); 5 Department of Oncology, La Croix du Sud Hospital, 31130 Quint Fonsegrives, France; esucsjl@club-internet.fr (E.S.); aplaurenty@capio.fr (A.-P.L.); 6 Department of Urology, CHU-IUC, 31000 Toulouse, France; roumiguie_mathieu@yahoo.fr; 7 Department of Urology, Bichat-Claude Bernard Hospital, Assistance Publique-Hopitaux de Paris, Paris University, 75018 Paris, France; evanguelosxylinas@hotmail.com

**Keywords:** bladder cancer, nodal disease, pN1, radical cystectomy, neoadjuvant, adjuvant, chemotherapy

## Abstract

Background: To compare overall survival (OS) outcomes in pN1-3 disease at the time of radical cystectomy (RC) for muscle invasive bladder according to the neoadjuvant chemotherapy (NAC) status. Materials and Methods: This multicenter study included 450 consecutive patients undergoing RC for muscle-invasive urothelial bladder cancer with pN1-3 pM0 disease from 2010 to 2019. NAC consisted in platinum-based chemotherapy. The primary endpoint was the comparison between NAC and non-NAC in terms of death from any cause. OS was assessed using the Kaplan–Meier method and multivariate Cox proportional hazards regression was used to estimate adjusted hazard ratios. Results: Median age was 69 years. Patients receiving NAC were younger (*p* = 0.051), and more likely had downstaging to non-muscle invasive disease (10.7% versus 4.3%, *p* = 0.042). Median OS was 26.6 months. NAC patients had poorer OS compared with those who did receive NAC (Hazard ratio (HR) 1.6; *p* = 0.019). The persistence of muscle-invasive bladder in RC specimens was also significantly associated with OS (HR 2.40). In the NAC cohort, the two factors independently correlated with OS were the number of positive lymph nodes (*p* = 0.013) and adjuvant chemotherapy (AC) (HR 0.31; *p* = 0.015). Conclusions: Persistent nodal disease in RC specimens after NAC was associated with poor prognosis and lower OS rates compared with pN1-3 disease after upfront RC. In this sub-group of NAC patients, AC was independently associated with better OS.

## 1. Introduction

Muscle-invasive bladder cancer is a highly aggressive disease with poor oncologic outcomes in case of lymph node involvement. Neoadjuvant chemotherapy (NAC) prior to radical cystectomy (RC) has proven to improve survival outcomes in localized muscle-invasive bladder [1,2,3]. Level I evidence demonstrates a survival advantage of 5% as well as complete response on both primary and nodal tumor tissues [3]. The pN0 rate after NAC in cN+ patients has been evaluated as high as 48% in a retrospective series of 304 patients [4]. However, in spite of this proven overall survival (OS) advantage, a certain proportion of patients did not respond to NAC and exhibited aggressive patterns at the time of deferred RC, including pN1-3 disease. Despite NAC, up to one-fifth of the patients harbored nodal disease involvement at the time of RC [5]. However, the differential outcomes of pN1-3 patients stratified by the use or not of NAC is not well established. Moreover, there is little evidence and no firm recommendation on how to treat patients with positive lymph nodes after RC, especially after NAC administration [6]. In that setting, the use of adjuvant chemotherapy (AC) and of platinum-based regimens could be limited by potential tumor cells resistance and cumulative toxicity. Thus, whereas the impact of NAC on survival outcomes of cN1-3 patients prior to RC has been assessed in retrospective trials, to our knowledge, no series has compared OS between NAC and non-NAC patients harboring pN1-3 disease at the time of RC, and therefore the potential benefit of AC administration in that setting [4,7]. Studies comparing oncologic outcomes of pN1-3 disease according to the NAC status are biased by the selection, in the NAC group, of patients who did not respond to chemotherapy given persistent or progressing node disease after NAC. This selection bias based on resistance to neoadjuvant therapy has to be considered but helped to understand the need for aggressive post-RC treatment or monitoring in case of NAC failure.

## 2. Materials and Methods

### 2.1. Patients

We included 450 consecutive patients that underwent radical cystectomy (RC) for muscle-invasive urothelial bladder cancer with pathologically proven nodal disease from 2010 to 2019 at two institutions. After institutional review board approval (IRB number: 00006477 2017-016; review board: CEERB Paris Nord), all patients gave their written informed consent to participate in the prospective assessment of the outcomes (personal data collection and analysis). All RC were planned for cT2-4 cM0 disease, and we only included patients with pN1-3 disease. Clinical stage showed cT3 and cT4 disease in 31% and 20% of NAC patients, and 30% and 13.8% of non-NAC patients, respectively (48.2% of missing data for that variable). Patients with distant metastases (pM1a-b) on the pre-operative computerized tomography (CT) scan were excluded from analysis. The CT scan was systematically performed at the time of diagnosis. RC was performed less than 6 weeks after the diagnosis or less than 6 weeks after the last cycle of NAC. In case of NAC, another CT scan was performed before RC to confirm the absence of progression during NAC which would contra-indicate surgery. NAC and AC consisted of platinum-based chemotherapy. All patients treated by NAC received MVAC (methotrexate-vinblastine-doxorubicine-cisplatin) or GC (gemcitabline-cisplatin) regimen. AC was defined as a chemotherapy regimen given after RC before any sign of post-surgery progression, and platinum-based chemotherapy was the regimen of choice in the absence of contra-indication. Chemotherapy regimen and number of cycles were administered at clinician discretion in accordance with institutional standards and on individual decision-making. Patients treated with adjuvant radiotherapy or a combination of radiation and chemotherapy were excluded. All pathology data, including TNM stage, tumor grade, presence of positive soft tissue margin, total number of removed lymph nodes (LN), and number of LN+ were obtained from the pathological reports. Clinicopathological characteristics, surgical and adjuvant treatments, and follow-up data were collected in medical records. The chemotherapy status (NAC, AC) was recorded.

### 2.2. Primary and Secondary Endpoints and Statistics

The primary endpoint was the comparison between NAC and non-NAC in terms of death from any cause. Overall survival (OS) was assessed from the date of surgery until the date of death. OS was estimated using the Kaplan–Meier method and was compared using log-rank analysis. OS rates were calculated with 95% confidence intervals. Multivariate Cox proportional hazards regression was used to estimate adjusted hazard ratios with 95% confidence interval. The limit of statistical significance was defined as *p* < 0.05. The SPSS 22.0 (IBM, Chicago, IL, USA) software was used for analysis.

## 3. Results

### 3.1. Clinical and Pathological Features of the Entire Cohort (n = 450) 

Median age was 69 years with 73.1% male patients (Table 1). Downstaging to non-muscle invasive disease in RC specimens was 5.0%. Lymphovascular invasion and concomitant carcinoma in situ (CIS) were reported in 67.1% and 40.2% of cases, respectively. Soft tissue surgical margins were positive in 12.9% of the specimens. Median lymph node yield and positive lymph nodes were 16 and 2, respectively. Overall, 12.4% and 54.2% of patients received NAC +/− AC, and AC only, respectively. Among the overall cohort, 4.4% of patients received both chemo regimens. Approximately, half of patients died after a mean follow-up of 23 months. Distant systemic progression (bone and/or visceral metastases) was reported in 41.8% of patients.

### 3.2. Comparisons of Clinical and Pathological Features Stratified by NAC Administration 

Clinical and pathological features of both cohorts were compared (Table 2). Patients receiving NAC were younger (65 versus 68 years, *p* = 0.051), and more likely had downstaging to non-muscle invasive disease (10.7% versus 4.3%, *p* = 0.042). No significant difference was seen regarding CIS, lymphovascular invasion, positive lymph nodes, and soft tissue margin. Non-NAC patients were more frequently treated by AC (56.9% versus 35.7%, *p* = 0.003) and developed fewer systemic progression (39.1% versus 60.1%, *p* = 0.002).

### 3.3. Survival Analysis in the Overall Cohort

**Figure 1:**
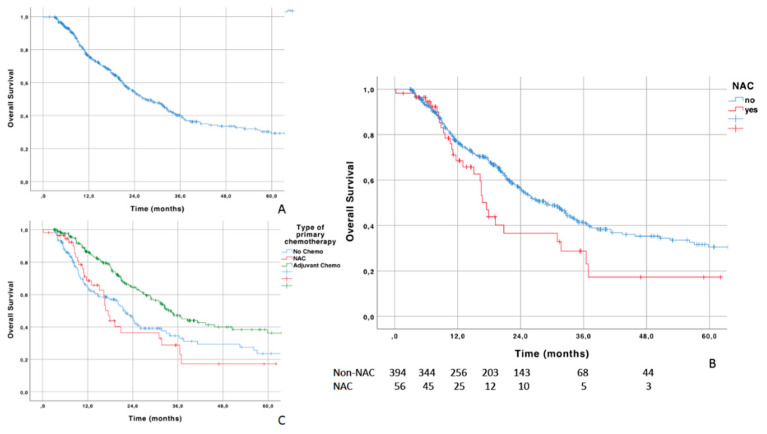
(**A**) Overall survival (OS) curve in the overall cohort; (**B**) OS stratified by the use of neoadjuvant chemotherapy (NAC); (**C**) OS stratified by the type of primary chemotherapy: neoadjuvant chemotherapy (NAC), adjuvant chemotherapy (AC), no chemotherapy.

The OS curve of the overall cohort is shown in Figure 1A. Median OS was 26.6 months. The 1-, 2-, and 5-year OS rates were 75.9% (±2.1), 54.3% (±2.7), and 29.2% (±3.2) in the overall cohort.

NAC patients had poorer OS compared with those who did not receive NAC (log rank test: *p* = 0.019, Figure 1B). The 1-, 2-, and 5-year OS rates were 66.8% (±7.3), 34.6% (±8.3), and 16.3% (±7.7) in the NAC cohort, versus 76.9% (±2.2), 56.3% (±2.8), and 30.5% (±3.5) in the non-NAC cohort. Median OS in the NAC and non-NAC cohorts was 16.7 and 28.8 months, respectively.

The OS curves were then stratified according to the type of primary chemotherapy received (Figure 1C): no chemotherapy, NAC, or AC. Patients treated by AC had better OS outcomes compared with those receiving NAC or no chemotherapy (log rank test: *p* < 0.001). Median OS was 33.6 months, compared with 22.0 and 16.7 months in the no chemotherapy and NAC cohorts, respectively. Survival curves did not differ significantly between patients who did not receive any chemotherapy and NAC patients, in spite of a trend toward better outcomes during the first 18 months after RC (*p* = 0.557). Curves crossed at this time point with better long-term outcomes in patients without any neoadjuvant or adjuvant chemotherapy regimens.

### 3.4. Multivariable Analysis of Predictive Factors for OS in the Overall Cohort

Cox regression model confirmed that NAC was independently associated with overall mortality (Table 3). NAC patients had a 1.6-fold higher risk of death compared with non-NAC patients (*p* = 0.018; 95% confidence interval: 1.09–2.47). The persistence of muscle-invasive bladder in RC specimens was also significantly associated with OS (HR 2.40; 95% confidence interval: 1.06–5.44). This negative effect of NAC (*p* = 0.072) failed to reach significance when AC was taken into the multivariable model. AC was then positively and independently correlated with improved OS (HR 0.56; 95% confidence interval: 0.42–0.73; *p* < 0.001).

### 3.5. Stratified Survival Analysis in NAC Cohort

**Figure 2:**
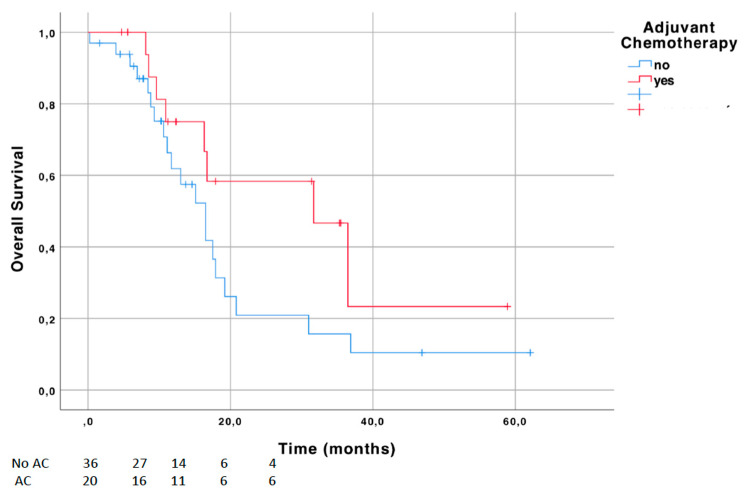
Survival curves for overall survival (OS) in the neoadjuvant chemotherapy (NAC) cohort stratified by the use of adjuvant chemotherapy (AC).

Among NAC cohort, the administration of adjuvant chemotherapy was correlated with improved OS, without significant difference (Figure 2; *p* = 0.099). Median OS was 16.5 versus 31.7 months in patients receiving AC after NAC. The one-year OS rates were 61.9% (±9.7) versus 75.0% (±10.8) comparing patients who received AC and those who did not.

### 3.6. Multivariable Analysis of Factors Associated with Overall Mortality in the NAC Cohort 

Cox regression analysis was performed in the subgroup of NAC patients (Table 3). Given the low number of patients (*n* = 56) and consequently the low number of events, we only included three factors which were the most correlated with overall mortality in univariable analyses. In the NAC cohort, the two factors independently correlated with overall mortality were the number of positive lymph nodes (>3 nodes; *p* = 0.013) and the administration of AC. AC was independently associated with a lower risk of overall mortality (HR 0.31; 95% confidence interval: 0.12–0.80; *p* = 0.015).

## 4. Discussion

NAC prior to RC has proven to improve survival outcomes in localized and locally advanced muscle-invasive bladder [1,2,3]. However, a non-negligible proportion of patients did not respond to NAC and exhibited aggressive patterns at the time of deferred RC including one-fifth of patients with nodal disease [5].

To date, there is little evidence on how to treat patients with positive lymph nodes after NAC and RC [6]. In a recent UK survey, 45% of oncologist responders would not give AC in patients with node disease after NAC and RC. Due to several factors, such as post-operative complications, impaired renal function, and poor performance status, the delivery of AC may be challenging even if an OS benefit is achieved [8]. Thus, the feasibility of re-challenging this group of NAC patients with AC is currently not well established, and patients are often offered salvage chemotherapy only at time of disease progression for palliation. A previous study of 37 patients with node positive disease after NAC previously suggested that patients who have persistent nodal disease have a very poor prognosis [9]. The two-year OS survival rate was 20%. The findings of this single-arm retrospective study highlighted a potential benefit from adjuvant chemotherapy. As reported in our series, there was a trend toward improved OS when AC was used.

While the rate of pT0 disease after NAC has been well assessed in the literature (approximately 30%), the complete response rate in node cannot be accurately evaluated due to the inaccuracy of preoperative evaluation. Indeed, node staging is currently performed by CT scan or pelvic magnetic resonance imaging (MRI). Both procedures are limited by poor sensitivity and specificity. In a series of clinical node-positive patients prior to NAC, Hermans et al. suggested that the rate of complete post-NAC response in pelvic lymph nodes (pN0) was 31% and 19% in cN1 and cN2-3 patients, respectively [7]. A complete response in lymph nodes has been evaluated at 48% in another retrospective study [4]. We were unable to assess this node downstaging rate given that we only included pN1-3 patients. However, even in patients having an aggressive disease with positive nodes at RC, our study suggests a positive impact of NAC on tumor tissue given that the pT0-1 rate was 10.8% in the NAC cohort, versus 4.3% only in non-NAC patients (*p* = 0.042). Unfortunately, given the limitations already evoked, the potential difference of response between primary cancer and metastatic nodal tissue cannot be relevantly evaluated.

The poorer OS achieved by NAC versus non-NAC patients with pN1-3 disease confirmed the need for adapting post-RC treatment in this high-risk sub-population. These patients will more frequently develop post-RC systemic progression (60.7% versus 39.1%) and die prematurely. Our findings suggest that the use of AC could be beneficial even after NAC. Indeed, OS was improved when AC was given, and AC was an independent protective factor in multivariable analysis, after taking into account positive lymph node burden and pT stage.

Consistently with French habits, MVAC was regarded in our experience as the first-line treatment of choice [10]. The pathological complete response rate achieved by dose dense MVAC appeared better than GC in retrospective studies [11]. Few patients received GC which could be preferred in other centers and/or countries due to a better toxicity profile. Comparable efficacy of GC has been emphasized, but in the metastatic setting [12]. Preliminary data from the VESPER trial (NCT01812369), comparing GC and MVAC as NAC, were presented recently, and the mature publication is awaited.

The role of AC after RC remains controversial. The main data come from underpowered trials due to poor recruitment, or from studies suffering from methodological issues. The advent of NAC before RC has also had a negative impact on enrollment in such trials [13]. The European Organisation for Research and Treatment of Cancer (NCT 30994) evaluated four cycles of immediate adjuvant chemotherapy versus six cycles of deferred chemotherapy at the time of relapse [14]. The benefit in OS was only seen in a small sub-group of pN0 patients (*n* = 86). Meta-analyses tend to confirm the reduction in the risk of death with AC (approximately 23%) [15,16]. Thus, although AC is no longer recommended, evidence suggests that it could be efficient, but mainly in chemotherapy-naive patients with locally advanced bladder cancer (pT3-4, pN0/pN +, pM0). Until now, no prospective trial has compared the sequence NAC versus NAC plus AC in patients with persistent locally advanced bladder cancer or lymph node involvement at the time of RC.

We did not report the detailed chemotherapy regimens in terms of number of cycles, toxicity data, palliative chemotherapy, and number of subsequent lines. The OS we showed could be impacted by all these parameters. Subsequent therapies for metastatic disease, that may have affected OS rates, were not available for all patients. Until recently, the only licensed second-line chemotherapy was vinflunine, which has demonstrated a three-month survival benefit with toxicity. However, the therapy landscape of advanced bladder cancer rapidly evolves. It is also worthy to note that this cohort was followed before the approval of immunotherapy regimens in advanced bladder cancer. The implementation of immunotherapy in the metastatic as well as in the neoadjuvant setting may modify the response to neoadjuvant treatment, as well as progression-free and overall survival [17]. In this study, we found that NAC patients treated by AC after RC achieved better OS outcomes compared with patients receiving only palliative chemotherapy. However, only one-third of NAC patients received AC due to poor performance status, post-operative complications, cumulative toxicity or various reasons. The possibility to change AC for adjuvant immunotherapy could increase the number of NAC patients eligible for adjuvant therapy and offer life-prolonging drug options in that particular setting of pN1-3 NAC patients.

The combination of therapy could also be an interesting option in pN1-3 disease. Zaghloul et al. recently demonstrated in a phase II study that the addition of radiotherapy to AC could improve the locoregional recurrence-free survival [18]. The trend reported in terms of OS has to be confirmed in larger phase III trials. The GETUG-AFU 30 trial (NCT03333356) is ongoing to evaluate the benefit of adjuvant radiotherapy in high-risk cancers in terms of pelvic recurrence-free survival as primary endpoint, and OS as secondary endpoint.

It seemed worthy to note that we only included in this study NAC patients who did not respond to chemotherapy given persistent or progressing node disease after NAC. This sub-group selection based on first therapy resistance explained the worse prognosis of NAC patients compared with non-NAC patients who were not selected by any type of treatment resistance. This selection bias has to be considered and helps to understand the need for aggressive post-RC treatment or monitoring in case of NAC failure.

Finally, the main limitation was the difficulty to draw any firm conclusion based on a retrospective study. In addition to potential selection biases in the selection of patients for NAC, for surgery and for AC, our results could have also been limited by the relatively small sample size. Currently, it is not possible to establish with absolute certainty what is the best sequence of perioperative treatments. However, to our knowledge, this study was the first to directly compare contemporary outcomes after RC in pN1-3 patients treated or not with NAC, and it confirmed the potential of AC even in patients already treated by NAC.

## 5. Conclusions

Persistent nodal disease in RC specimens after NAC is associated with poor prognosis and lower OS rates compared with pN1-3 disease after upfront RC. In this sub-group of NAC patients, AC was given to one-third of NAC patients and was an independent predictive factor for better OS outcomes. Larger prospective data as well as studies assessing the impact of other adjuvant therapies such as immunotherapy or radiotherapy are awaited.

## Figures and Tables

**Table 1:** Overall cohort clinical and pathological characteristics (*n* = 450).

	*N* = 450
Gender (*n*, %):	
Male	329 (73.1)
Female	121 (26.9)
Age (years):	
Mean	67.5
Median (range) IQR	69.0 (25–93)
Pathological stage (*n*, %):	
pT0-pTis	12 (2.6)
pT1	11 (2.4)
pT2	78 (17.3)
pT3	247 (54.9)
pT4	102 (22.7)
Presence of lymphovascular invasion (*n*, %)	302 (67.1)
Presence of concomitant CIS (*n*, %)	181 (40.2)
Presence of soft tissue surgical margins (*n*, %)	58 (12.9)
Number of lymph nodes analyzed:	
Mean	17.5
Median (range) IQR	16.0 (1–70)
Number of positive lymph nodes:	
Mean	3.9
Median (range) IQR	2.0 (1–41)
Type of chemotherapy regimen (%):	
None	170 (37.8)
Neoadjuvant without adjuvant	36 (8.0)
Neoadjuvant + adjuvant	20 (4.4)
Adjuvant only	224 (54.2)
All-cause death (%)	220 (48.9)
Follow-up (months):	
Mean	23.0
Median (range) IQR	17.3 (3–130)

IQR = interquartile range, CIS = carcinoma in situ.

**Table 2:** Comparisons between neoadjuvant chemotherapy (NAC) and non-NAC patients.

	NAC Cohort	Non-NAC Cohort	*p*-Value
	*N* = 56	*N* = 394	
Male (%) gender	46 (82.1)	283 (71.8)	0.103
Age (mean)	65.0	68.0	0.051
Pathological stage (%):			0.097
pT0-pTis	3 (5.4)	9 (2.3)	
pT1	3 (5.4)	8 (2.0)	
pT2	6 (10.7)	72 (18.3)	
pT3	29 (51.8)	218 (55.3)	
pT4	15 (26.8)	87 (22.1)	
Previous history of non-muscle-invasive bladder tumor before T2-4 diagnosis (%)	6 (10.7)	17 (4.3)	0.042
Presence of lymphovascular invasion (%)	42 (75.0)	260 (66.0)	0.179
Presence of concomitant CIS (%)	17 (30.4)	164 (41.6)	0.108
Soft tissue surgical margins (%)	7 (12.5)	51 (12.9)	0.926
Number of lymph nodes analyzed yield (mean)	17.6	17.1	0.777
Number of positive lymph nodes (mean)	3.8	4.8	0.197
Adjuvant chemotherapy administration (%)	20 (35.7)	224 (56.9)	0.003
Distant metastases (%)	34 (60.7)	154 (39.1)	0.002

NAC = neoadjuvant chemotherapy, CIS = carcinoma in situ.

**Table 3:** Multivariable Cox regression analyses for predictors of overall survival (OS) in the overall cohort and in the neoadjuvant chemotherapy (NAC) cohort.

	HR	95% CI	*p*-Value
Overall cohort			
Model 1			
Gender	0.884	0.647–1.209	0.441
Age (continuous)	1.009	0.996–1.023	0.178
Muscle-invasive disease	2.404	1.062–5.442	0.035
Lymphovascular invasion	0.882	0.664–1.171	0.385
Concomitant CIS	1.088	0.830–1.427	0.540
Soft tissue surgical margin	1.338	0.910–1.965	0.138
Positive lymph nodes >3	1.283	0.959–1.717	0.093
NAC	1.638	1.089–2.465	0.018
Model 2			
NAC	1.445	0.968–2.159	0.072
Adjuvant Chemotherapy	0.557	0.426–0.728	<0.001
NAC cohort			
Muscle-invasive disease	0.296	0.060–1.470	0.137
Positive lymph nodes >3	3.281	1.287–8.365	0.013
Adjuvant chemotherapy	0.310	0.120–0.800	0.015

HR = hazard ratio; CI = confidence interval; CIS = carcinoma in situ.
